# Evaluation of Low-Intensity Pulsed Ultrasound on Stress Fractures to Reduce the Time to Return to Sport or Activity in the Physically Active Population: A Systematic Review

**DOI:** 10.7759/cureus.49129

**Published:** 2023-11-20

**Authors:** Mackinzie McDaniel, Nicholas R Eltman, Jason Pan, Randel L Swanson

**Affiliations:** 1 Physical Medicine and Rehabilitation, University of Pennsylvania Perelman School of Medicine, Philadelphia, USA; 2 Center for Neurotrauma, Neurodegeneration and Restoration, Cpl. Michael J. Crescenz VA Medical Center, Philadelphia, USA; 3 Physical Medicine and Rehabilitation, Rowan-Virtua School of Osteopathic Medicine, Stratford, USA

**Keywords:** return to activity, low intensity pulsed ultrasound (lipus), stress fracture, athletic injury, athlete

## Abstract

Stress fractures are a common and significant source of pain and burden that can require long periods of rest from physical activity to allow adequate healing. Specifically in athletes or those with physically demanding occupations, the prolonged period of rest and the potential for requiring surgical intervention in the case of poor or delayed healing can have devastating impacts on these individuals’ careers and socioeconomic well-being. In this population, successful healing, in addition to a quicker healing time and a sooner return to activity, are important outcomes when faced with treating stress fractures. The use of low-intensity pulsed ultrasound (LIPUS) to accelerate bone healing has been a topic of investigation, though little research has explored the use of LIPUS specifically in the physically active population. The purpose of this study was to review the existing literature on the use of LIPUS for stress fracture healing in the physically active population with the outcome of a quicker return to sport or physical duties.

The PubMed and Embase databases were screened for relevant articles using defined Preferred Reporting Items for Systematic Reviews and Meta-Analyses (PRISMA) criteria. Two independent researchers screened articles using PICOS criteria for inclusion in the review. Data were independently extracted regarding study and population characteristics as well as outcome measures, including time to healing of fractures and time to return to sport or physical activity.

Five studies were ultimately included in the systematic review. One study investigated the use of LIPUS in pediatric athletes with spondylolysis, while four studies investigated lower extremity stress fractures in adult populations. All reported the outcomes of either rate or time to healing and ability to return to sport or activity. One study found a statistically significant improvement in the rate of bone union in the intervention group undergoing LIPUS compared to the control. Two studies found a statistically significant decrease in the time to resolution of symptoms, allowing an earlier return to sport or physical duties. Two studies found no difference in the time to healing or success rate of healing between the LIPUS group and the control group.

This review of the literature suggests that the use of LIPUS for the treatment of stress fractures in the athletic or physically active population has the potential to expedite the resolution of symptoms and return to activity. Due to the heterogeneity of the existing studies, more research is needed to definitively determine the most appropriate clinical application of LIPUS and its most effective ultrasound settings. Further research should be directed toward more controlled studies specifically investigating the athletic and physically active population.

## Introduction and background

Stress fractures represent a large proportion of activity-limiting injuries in the physically active population, including athletes of all levels as well as military personnel. It has been reported that up to 80% of all duty-limiting injuries among US military members are lower extremity overuse injuries, including stress fractures [1]. In the sporting community, it is estimated that 10% of all sport-related injuries are stress fractures, 90% of which occur in the lower extremities [2]. Similarly, stress fracture of the pars interarticularis of the lumbar vertebrae of the spine, known as spondylolysis, affects the athletic and active community at an alarming rate. Studies have found that as many as 60% of athletes’ low back pain is secondary to spondylolysis [3].

When diagnosed with a stress fracture, individuals considered low risk of delayed healing are treated conservatively with periods of rest and restricted or non-weight-bearing status (ranging from weeks to months) followed by a gradual return to activity, while individuals at higher risk of delayed or non-union may be faced with surgical intervention [2]. When formulating a treatment plan, providers must consider the trauma of undergoing surgical procedures, especially in an otherwise healthy and active population. Given the physical, economical, and emotional burden of long periods of delayed participation in sporting activities or duties, there is a need to explore alternatives when treating bone stress fractures. The use of low-intensity pulsed ultrasound (LIPUS) represents a unique opportunity for this active population to avoid surgical options while also potentially shortening conservative treatment to achieve the quickest, safest return to sport and activity with the best healing outcome.

This review aims to determine if the use of LIPUS therapy accelerates healing and reduces the time to return to sport or activity for active individuals diagnosed with stress fractures. Much of the current research has focused on the use of LIPUS in the case of non-union or mal-union fracture healing, typically in populations with serious co-morbidities or sedentary lifestyles. A recent systematic review that was performed on 23 randomized controlled trials investigating the use of LIPUS to accelerate radiographic healing of fresh fractures found that LIPUS did indeed shorten the time to evidence of radiographic healing [4]. These results have been duplicated in several reviews over the past few decades, including some focused specifically on its use as an alternative to surgery for fractures that had failed to heal properly with time and rest [5]. Such results hold promise for expanding the use of LIPUS in stress fracture healing; however, few investigations have focused on the use of such technology in the otherwise healthy and active population, with a focus on recreational or professional athletes or physically demanding professions, and with return to physical function as an outcome measure. Given the paucity of these data, we aim to review the available research in order to provide evidence-based information on how to best treat this population when presenting with a stress fracture. To the best of our knowledge, no review specifically investigating this population yet exists. We hypothesized that the use of LIPUS in this population would accelerate bone healing and return to sport or activity.

## Review

Methods

Search Strategy

A literature search was conducted using two databases: PubMed and Embase. A total of 1072 unique articles were found using the keyword search terms "low-intensity pulsed ultrasound, “low-intensity pulsed ultrasound”, “stress fracture, healing”, or “athlete or sport.”

Eligibility Criteria

The Participant, Interventions, Comparisons, Outcomes, and Study Designs framework (PICOS) was utilized to determine article eligibility for inclusion in the review [6]. The specific PICOS criteria used were as follows.

Participants: Athletes or active individuals presenting with a stress fracture were included. No age restriction or sport/activity restriction was used.

Intervention: LIPUS was used.

Comparison: Placebo/sham ultrasound treatment or conventional conservative treatment

Outcome measures: Time to return to physical activities of either sport or physical duties; radiographic or clinical evidence of fracture healing was studied.

Study design: Randomized controlled trials, prospective cohort studies, or retrospective cohort studies published between 1973 and June 2023 were included. Narrative reviews full-length, letters or opinion articles, case series or studies, abstracts or poster presentations, and studies not available in full-reviews full-length English text were excluded.

Study Screening

All stages of screening and data extraction were completed within the Covidence systematic review software [7]. Articles resulting from the literature database search were uploaded to Covidence and first screened by title and abstract by two independent reviewers to progress to the full-text review stage. Articles that appeared relevant to the topic at hand were retrieved in their full-text format and, again, evaluated by two independent reviewers to determine whether they were eligible for inclusion in the final review. Any disagreement between reviewers was resolved through discussion. Inclusion was based on the PICOS criteria as detailed above.

Data Abstraction

**Figure 1:**
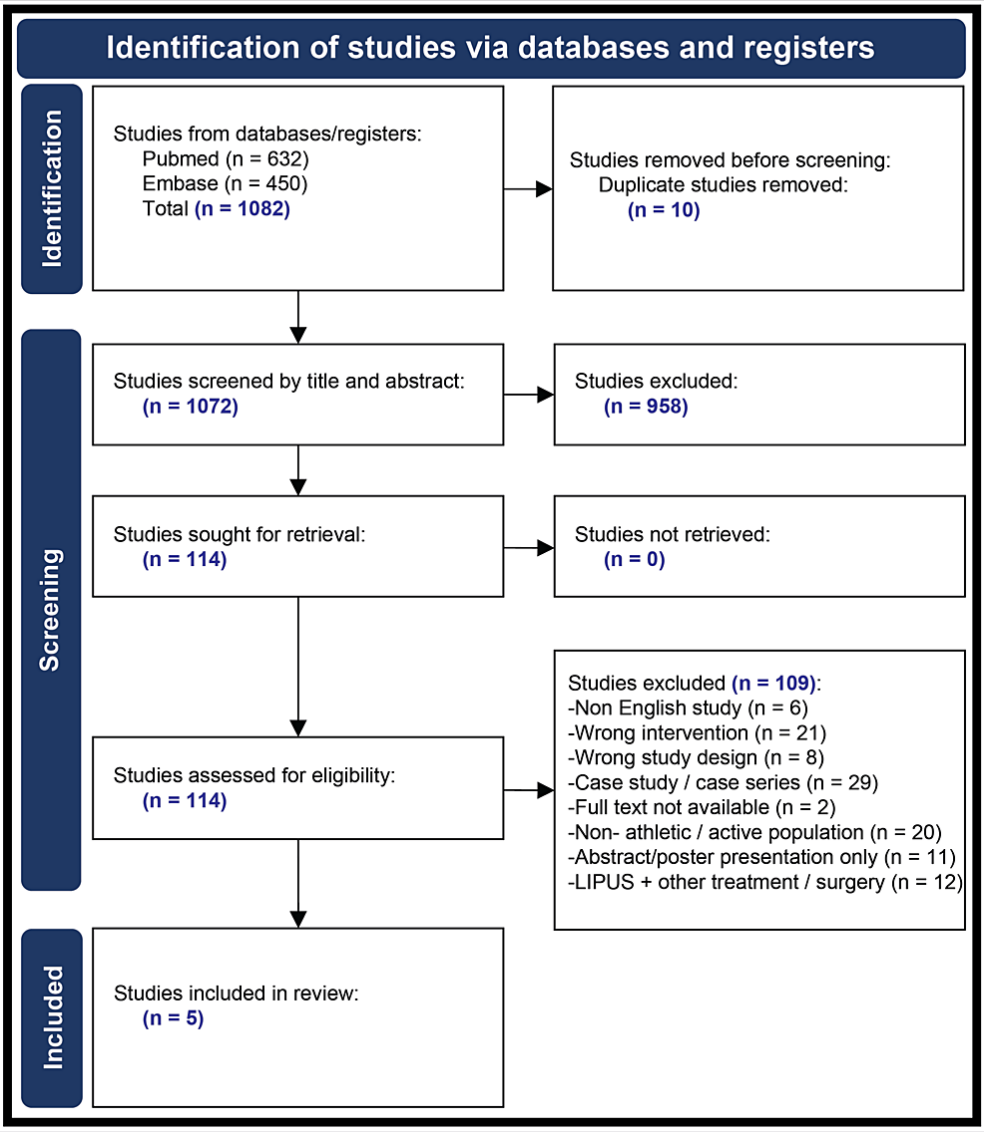
PRISMA flow diagram showing the selection process of reviewed articles PRISMA: Preferred Reporting Items for Systematic Reviews and Meta-Analyses

Two reviewers independently extracted data from the included studies. Data extracted included author, publication year, study design, sample size, patient demographics, intervention(s), outcome measure(s), outcome measure result(s), study limitations, and author conclusions. After completion, both independent data extractions were assigned within Covidence to the primary reviewer for consensus. Any disagreements within the extracted data between each reviewer were resolved by discussion or by an independent third party if necessary for a final determination. The Preferred Reporting Items for Systematic Reviews and Meta-Analyses (PRISMA) flow diagram below (Figure 1) details the specifics of the study screening and selection process.

Results

Study Characteristics

Five studies were found to meet the PICOS criteria and included in the final review (Table 1), comprising three randomized controlled trials [8-10], one non-randomized controlled trial [11], and one prospective cohort study [12]. All studies compared the use of low-intensity pulsed ultrasound to a conservative therapy. Two studies focused specifically on tibial stress fractures [9,10], while one included tibia, fibula, and femur stress fractures [11], one looked at stress fractures of tibia, fibula, and metatarsals [8], and one investigated stress fractures of the pars interarticularis of the lumbar spine, specifically at the L4 or L5 levels [12] (Table 2). The duration of the studies varied, with some giving fixed timelines to conclude the testing while others varied based on radiographic and subjective findings from the patient, with an overall range of one to five months.

**Table 1:** Study characteristics LIPUS: low-intensity pulsed ultrasound; CG: control group

Reference	Study design	Purpose	Duration	Number of Participants
Gan et al. [8]	Double-blinded, randomized, placebo-controlled trial	To determine the effectiveness of LIPUS for the improvement of lower limb bone stress injuries	12 weeks	23 total: 10 LIPUS, 13 CG
Yadav et al. [9]	Double-blinded, randomized, placebo-controlled trial	To determine if use of LIPUS reduced the time to return to training in military recruits with tibial stress fractures	Time until resolution of symptoms or return to duty (max recorded 55 days); patients followed for one additional month after return to training	67 total: 39 in LIPUS, 28 in CG
Rue et al. [10]	Double-blinded, randomized controlled trial	To determine whether LIPUS reduces tibial stress fracture healing time	Dependent on duration of symptoms (average 45 days)	26 total: LIPUS 14, CG 12
Yadav [11]	Non-randomized controlled trial	To determine if LIPUS therapy speeds healing of stress fractures and reduces time to return to training	4-8 weeks dependent on symptoms	75 total: 32 CG, 43 LIPUS
Arima et al. [12]	Prospective cohort study	To determine if the application of LIPUS accelerates healing in the progressive stage spondylolysis and if there are adverse events related to LIPUS treatment	Until confirmed bone union/non-union via checkups every 1.5 months (max 5 months to determine nonunion)	13 total: 7 CG and 6 LIPUS

**Table 2:** Participant characteristics LIPUS: low-intensity pulsed ultrasound; CG: control group

Reference	Age (years)	Activity level or type	Fracture type/location	Time since injury	Gender
Gan et al. [8]	Overall: 30.4 ± 12.1, LIPUS: 32.7 ± 10.6, CG: 28.6 ± 13.3	All reported to be active at a recreational to elite level; a specific numerical breakdown of distribution of activity levels was not provided	Overall: 11 tibial, 5 fibula, 7 metatarsal; LIPUS: 5 tibial, 2 fibula, 3 metatarsal; control group: 6 tibial, 3 fibula, 4 metatarsal	Time since injury not recorded; time zero determined to be time of presentation with symptoms	Overall: females 19, males 4; LIPUS: females 7, males 3; CG: females 12, males 1
Yadav et al. [9]	Groups were matched for age, with no significant difference between groups reported, but age not specifically given	Military recruits in active training	Tibial	Not reported; groups reported to be matched for delay from symptom onset to diagnosis	Not documented
Rue et al. [10]	LIPUS: 18.6 ± 0.8, CG: 18.4 ± 0.8	Naval Academy Initial Training Camp	Tibial	Measured by days since start of symptoms to diagnosis of stress fracture: LIPUS: 25.6 ± 12.7, CG: 21.7 ± 6.7, non significant	Overall: 13 male, 13 female, LIPUS: 7 male, 7 female, CG: 6 male, 6 female
Yadav [11]	17 to 21 for all participants	Military recruits in basic training camp	Control group: tibia 23, fibula 6, femur 1, both tibia and fibula 2, LIPUS group: tibia 39, fibula 1, femur 3	Not measured	Not documented
Arima et al. [12]	14.6 ± 2.5 for all participants	Not defined, all participants were pediatric athletes	Pars interarticularis of the lumbar spine, specifically at the L4 or L5 levels	Fresh fracture as determined by high signal change on MRI	Overall: 76.9% male, LIPUS: 66.7% male, CG: 85.7% male

Participant Characteristics

The age of participants differed by study (Table 2). Arima et al. focused on a pediatric population with a mean age of 14.6 ± 2.5 years [12]. Gan et al. investigated a population more advanced in age than the other included studies, with a mean age of 30.4 ± 12.1 years [8]. Rue et al. and Yadav both looked at an age group of approximately 17 to 21 years [10,11]. Finally, Yadav et al. did not report a specific age group but reported that groups were matched by age [9]. All studies included individuals defined as physically active or athletes, ranging from recreational athletes to military recruits in training (Table 2).

Interventions and Specific Ultrasound Settings

Table 3 reflects the inclusion, exclusion, intervention, ultrasound settings, and control group for each study. The characteristics of the ultrasound were not uniform across all studies, and specific details were not uniformly reported. Both Yadav and Yadav et al. used the same power (1 Watt/sq.cm), though Yadav did not list a more specific setting, and Yadav et al. used a higher setting than all other studies at 3 MHz [9,11]. Arima et al. and Gan et al. both used similar settings of 1.5 MHz waves with 1 kHz frequency bursts at 200 m/s [8,12]. The interventions in three studies included an experimental group of 20 minutes of LIPUS daily in addition to the conservative treatment of the control group. Yadav used five minutes of daily ultrasound [11], and Yadav et al. used 10 minutes daily [9]. Control groups varied, with most being the standard of care with conservative rest, and three studies included equal amounts of time undergoing sham ultrasound treatment as the experimental group.

**Table 3:** Study inclusion/exclusion criteria and intervention details *Exams used included positive fulcrum test, percussion sign and tuning fork test; **clinical signs included tenderness, swelling, edema, positive fulcrum test, percussion test and one leg hop. MHz: Megahertz; ms: millisecond; kHz: killohertz; W/cm^2^: Watts per centimeter squared; CT: computed tomography scan; MRI: magnetic resonance imaging; PT: physical therapy; BSI: bone stress injuries.

Reference	Inclusion criteria	Exclusion criteria	Intervention	Intervention ultrasound settings	Control
Gan et al. [8]	Grade 2-4 BSI on MRI of the tibia, fibula or 2nd–4th metatarsal; sporting level of recreational through elite athlete	Subjects with other lower limb BSI	20 minutes of LIPUS daily for 28 days	1.5 MHz in burst of 200 ms at a frequency of 1 kHz with average intensity of 117% ± 30% mW/cm^2^	20 minutes daily of sham LIPUS with ultrasound device for 28 days
Yadav et al. [9]	Military recruits with grade 2-3 tibial stress fractures diagnosed by radiograph + clinical signs**	Less than grade II stress fracture, stress fracture not in the tibia	10 minutes of LIPUS administered daily by blinded researcher until resolution of clinical signs + pain control with paracetamol and icing	3 MHz, power of 1 W/cm^2^, pulsed mode with a duty cycle of 50%	10 minutes daily of non-functioning ultrasound unit administered by blinded researcher +pain control with paracetamol and icing
Rue et al. [10]	Diagnosis of tibial stress fracture during 6 week initial Naval Academy training, completion of questionnaire	Stress fracture location other than tibia, not agreeable to follow up at clinic, other medical problems preventing use of daily ultrasound	20 minutes of LIPUS daily via Exogen system + standard of care	Not defined	Standard of care + 20 mins daily of sham LIPUS ultrasound
Yadav [11]	Stress fracture diagnosed by clinical history, exams* and radiographic findings; member of recruits undergoing training at artillery center Hyderabad from Nov 98 to May 99.	None specifically defined	5 minute LIPUS administered daily until meeting criteria for healing + pain control with Paracetamol and Icing + avoidance of running and jumping activities	1 W/cm^2^	Rest from running and jumping activities until resolution of symptoms + kept in hospital instead of daily activities
Arima et al. [12]	Progressive stage lumbar spondylolysis diagnosed on CT + MRI with high signal change	Patients without high signal change on MRI or not agreeable to completing the study	20 mins daily LIPUS applied by sonic accelerated fracture Healing system + conservative treatment	1.5 MHz waves in bursts of 200 ms at frequency of 1 kHz with average intensity of 60 mW/cm^2^	Conservative treatment with trunk brace + PT at hospital 2 times a month until completion of treatment

Outcomes: Time to Stress Fracture Healing and Time to Return to Activity

Time to heal and time to return to physical activities were reported in all studies, although sometimes indirectly. As seen in Table 4, primary and secondary outcome measures varied among the included studies. Healing with bone union as determined by imaging was directly reported by Arima et al., finding a statistically significant increase in bone union in the LIPUS group with a union rate of 66.7% versus 10% in the control group [12]. The average time from the start of treatment to bone union was also found to be statistically significantly shorter in the LIPUS group; however, time to actual resumption of sports was reported as "all subjects able to return to sports" without specific data or time frames [12]. Similarly, Gan et al. used imaging to report healing outcomes [8]. This study used MRI fracture grade and edema as a sign of healing and found a non-significant reduction of injury site edema between groups [8]. The number of symptomatic days with pain limiting return to activities was reported to be non-significantly different between groups, though no data were supplied. Rue et al., Yadav, and Yadav et al. all reported time to return to sport or duty directly, with Rue et al. finding a non-significant difference between groups and both Yadav and Yadav et al. reporting a statistically significant shorter time to return to duty in the LIPUS group [9-11].

**Table 4:** Study outcomes, conclusions, and limitations *Criteria for return to duty included pain-free activities of daily living (ADLs), no local tenderness or warmth, negative fulcrum test, and one leg hop without pain. LIPUS: low-intensity pulsed ultrasound; CG: control group; LBP: low back pain; MRI: magnetic resonance imaging; BSI: bone stress injury.

Reference	Primary outcome	Secondary outcomes	Conclusion	Major limitations
Gan et al. [8]	Change in MRI grade from week 0 to 12: LIPUS 2.2, CG 2.4, with a non-significant difference of 0.776; change in MRI edema (cm) at injury site LIPUS: 3, CG: 4.1, with nonsignificant difference of 0.271	The number of symptomatic days with night pain, pain with walking, sitting, hopping and running, and tenderness over the area not found to be statistically different	LIPUS did not improve healing of lower limb BSI in a general sporting, civilian population over conventional conservative therapy	Small population consisting mainly of older females, compliance not monitored with home units, weight bearing and activity level of subjects during treatment period not controlled for or recorded
Yadav et al. [9]	Average number of days of incapacitation (time to return to duty): LIPUS: 25.46 days, CG: 39.92 days, statistically significant	No secondary outcome measures reported	Low-intensity pulsed ultrasound treatment is effect in shortening the time to return to active duty in tibial stress fractures	No one-month follow-up data reported, did not use radiographic evidence for healing, specific population looking at only military recruits and only stress fractures, did not specify how the participants were recruited into the study or if activity throughout treatment was monitored or limited
Rue et al. [10]	Days of treatment: LIPUS: 27.5 ± 11.6 days, CG: 31.5 ± 13.2 days, not significant	Total days of symptoms (time to return to duty): LIPUS 56.2 ± 19.6 days, CG 55.8 ± 15.5 days, not significant	LIPUS did not significantly reduce the healing time of tibial stress fractures	Delay in the initiation of treatment for average of 29 days from start of symptoms, did not use optimal dosage of LIPUS (treatment daily vs the three times daily recommended by manufacturer)
Yadav [11]	Average total treatment duration: CG 75.3 days, LIPUS group 15.05 days, significant difference with p<0.001, with determination of readiness of return to duty by fulfilling predefined criteria*	Rate of failure to show improvement: Control 9.4%, LIPUS 9.3%, Reoccurrence of symptoms after resumption of training: Control 3.1%, LIPUS: 9.3%, overall success of treatment: Control 87.5%, LIPUS 81.4%	LIPUS therapy presented a safe, non-invasive modality of treating stress fractures that led to an early return to training duties	For patient safety, control group remained hospitalized, while LIPUS group returned to daily activities without training, lack of use of radiographic evidence for healing, no clear interval for re-evaluation of healing
Arima et al. [12]	Union rate of 10% in CG vs 66.7% in LIPUS, p=0.020; average treatment time to bone union (months): 3.8 GC vs 2.7 LIPUS	Reported subjective decrease in LBP in both CG and LIPUS; both LIPUS and GC reported ability to return to sports activity	LIPUS increased bone union in patients with progressive stage spondylolysis within a shorter duration	Small sample size and short follow up period, non-uniform type of brace, potential non-compliance with bracing

Discussion

Stress fractures represent a source of pain and frustration for athletes and active individuals of all levels. With prolonged healing times requiring periods of rest of varying lengths and time away from physical activities, stress fractures can have a significant impact on all facets of life for these individuals. Stress fractures, especially those of the lower extremity and the spine, are particularly insidious in nature, often leading to chronic courses due to delayed or failed healing and recurrence after healing [2]. Given this nature, the medical community has sought to find new ways to treat stress fractures in hopes of more successful and faster healing. To date, there has been little research investigating the use of LIPUS specifically in the athletic population. As with all medical treatments, it is important to consider the population being treated and their goals for treatment. Particularly in the athletic community, not only is bone healing important, but also the time to return to athletic activity and the duration of symptoms preventing return are significant issues. Considering these outcomes, this review examines the existing literature evaluating the efficacy of low-intensity pulsed ultrasound in the healing of stress fractures, with the goal of allowing an earlier return to sports and shortening the duration of symptoms.

Overall, when considering the outcome of time to return to activity, statistically significant results were found in three of the five studies [9,11,12], with the two remaining studies finding no difference between the control group and the LIPUS group [8,10]. Due to the few available studies investigating the LIPUS intervention specifically in the athletic population, there was a considerable amount of heterogeneity between study intervention protocols, population characteristics, and outcome measures, potentially influencing the lack of significant results in two of the studies [8,10].

Table 1 reflects the characteristics of each study. Of the five included studies, it is important to note that the commencement and duration of treatment from the onset of injury differed (as noted in Table 2). Previous research has noted the importance of the early initiation of LIPUS after injury as a crucial factor in its success, with most successful studies initiating treatment within seven days of injury or presentation [13]. Studies by Arima et al., Yadav, and Yadav et al. initiated treatment within an early timeframe and found statistically significant results [9,11,12]. In fact, stress fractures determined to be “fresh” by magnetic resonance imaging (MRI) were an inclusion criterion for Arima et al., and treatment was started immediately after detection [12]. In both Yadav and Yadav et al., treatment was initiated as soon as subjects presented with symptoms [9,11]. Though the exact timeline was not provided, given the daily, repetitive physical activity of military training, it is reasonable to assume that these subjects developed and sought attention for their symptoms more quickly than other populations that were not undergoing such a rigorous and condensed training schedule. In contrast, Rue et al. reported a delay in the initiation of treatment by an average of 29 days [10], and Gan et al. did not report the time to treatment start at all, though it can be assumed to be delayed as the participants were recruited for the study by referral from outpatient clinics, resulting in a potential period of waiting between appointments and the start date of treatment [8]. This discrepancy among studies and its potential relationship with significant findings represent an important gap in the existing knowledge of LIPUS use and how it can be best applied in real-world settings. In current clinical practice in the United States, Medicare insurance does not cover the cost of LIPUS treatment until the patient has demonstrated failure of conventional conservative treatment and is diagnosed with non-union as demonstrated by radiographic images at least 90 days apart, leading to a delay of weeks to months before treatment with LIPUS can be initiated [14]. More rigorous and well-controlled studies are needed to determine if the relationship between the time to start LIPUS and the success of LIPUS holds true and has the potential to impact access to beneficial stress fracture treatment for a great magnitude of individuals.

Additionally, gender is an important factor in the healing of stress fractures. Female athletes have been reported to experience high-risk stress fractures at a greater rate than their male counterparts and are more likely to experience difficulty with healing [15]. Notably, the study by Gan et al., which found no statistically significant difference in healing between groups, was a predominantly female population [8]. Of the studies that did find a significant difference, Arima et al. [12] had a predominantly male population of nearly 80%, and both the Yadav [11] and Yadav et al. [9] studies, while not directly reporting the gender distribution of their subjects, recruited participants from a training center in the Indian Army, which is reported to only be 0.56% female [16]. The difference observed between males and females in the development and healing of high-risk stress fractures is thought to be multifactorial in nature [15], which suggests future studies should evaluate these populations separately.

The difference in the overall study protocol, including ultrasound settings in the intervention group, is another discrepancy between the reviewed studies that could have led to differing results. The specific ultrasound settings used by the studies can be seen in Table 3. While Arima et al. and Gan et al. used the same settings and daily intervention duration, their studies are difficult to directly compare due to the different gender distribution of their populations and their investigation of spinal stress fractures versus lower limb stress fractures [8,12]. Notably, Yadav et al. used settings of a much higher frequency for a shorter duration per day [9], while Rue et al. and Yadav did not define their ultrasound settings in detail [10,11]. This variation and paucity of reported information highlight the need for more standardized trials involving LIPUS in athletic and/or active populations. Most current available literature reports the importance of using low-intensity settings in a pulsatile manner to achieve bone healing with ultrasound, citing the potential bone-damaging effects of higher frequencies [13]. Due to the inconsistency of reporting, it is not possible to ascertain what frequencies were used in all studies. Additionally, recommended settings differ by device manufacturer, another variable that was not constant across studies, though it should be noted that the settings used in both Arima et al. [12] and Gan et al. [8] are those most frequently reported in the existing literature [13].

This review highlights many of the limitations of the current research on the use of LIPUS in the treatment of stress fractures in athletic or active populations. Arima et al. lacked standardization in the treatment of the control group, with subjects receiving different types of back braces without a randomization protocol or documenting which subject received which type of brace [12]. None of the included studies monitored for compliance with physical activity restrictions during the treatment period, if any existed. This has a potential impact on the results of the studies, as the conventional standard of care treatment is restricted activity and rest until healing, but only in Yadav was the activity level of the control group actually monitored by keeping the control group hospitalized [11]. Meanwhile, the intervention group and the intervention groups of all studies included were allowed to resume their daily activities as desired with instructions to avoid regular training or sports. These uncontrolled settings make drawing a conclusion from the data provided difficult due to the many potentially confounding factors.

Moving forward, this review highlights the need for more uniform, directed research focusing on a standardized LIPUS protocol in athletes and/or active individuals with stress fractures. The heterogeneous nature of the included studies reviewed here makes it difficult to draw a conclusion as to whether LIPUS accelerates stress fracture healing for a quicker return to sport or activity. Considering that in all studies the subjects returned to sport or activity more quickly or, at the very least, in a time frame not significantly different from conservative treatment, the argument can be made that LIPUS remains a viable consideration for the treatment of stress fractures in active and/or athletic individuals when considered against conventional conservative treatment.

## Conclusions

A paucity of literature exists exploring the use of low-intensity pulsed ultrasound as a mechanism for speeding the healing of stress fractures in athletes and other active individuals. This review of the existing literature aims to better guide clinical practice for treating stress fractures in this patient population. The included studies suggest that LIPUS represents a potential treatment for a quicker return to sport or activity after a stress fracture in athletic and active populations, though more research is needed to fully evaluate how LIPUS might be best utilized in clinical practice. Given the low risk of LIPUS treatment and the potential benefit of earlier stress fracture healing and return to activity, LIPUS represents a viable alternative to conventional conservative treatment that should be investigated further.
